# Predictive factors for residual leg numbness after decompression surgery for lumbar degenerative diseases

**DOI:** 10.1186/s12891-022-05848-y

**Published:** 2022-10-13

**Authors:** Tao Zou, Hao Chen, Ping-Chuan Wang, Hui-Hui Sun, Xin-Min Feng

**Affiliations:** Department of Orthopedics, Clinical Medical College of Yangzhou University, No.98 Nantong West Road, 225001 Yangzhou, Jiangsu province China

**Keywords:** Numbness, Decompression surgery, Lumbar degenerative diseases, Predictive factors

## Abstract

**Background:**

The purpose of this study is to evaluate the change patterns of leg numbness (LN) after lumbar decompression surgery (LDS), and to find the predictive factors that affect the recovery of numbness.

**Methods:**

Patients who underwent LDS in our institution between August 2020 and July 2021 were prospectively enrolled in this study, and were followed by a 12-month follow-up. The degree of LN, leg pain (LP) and the disability were assessed using the visual analog scale (VAS) and oswestry disability index (ODI).

**Results:**

A total of 314 patients finished the 12-month follow-up. The preoperative mean VAS-LN score was 3.49 ± 2.44, which decreased to 1.91 ± 1.30 at 3 months, to 1.29 ± 0.97 at 6 months and to 1.26 ± 0.96 at 12 months after surgery. The preoperative mean VAS-LP score was 6.05 ± 1.30, which decreased to 2.00 ± 0.86 at 3 months, to 1.02 ± 0.80 at 6 months, and to 0.49 ± 0.71 at 12 months after surgery. The preoperative mean ODI score was 27.90 ± 7.08, which decreased to 9.73 ± 3.09 at 3 months, to 6.72 ± 2.98 at 6 months, and to 4.57 ± 2.76 at 12 months after surgery. Via multivariate logistic regression analysis, only preoperative VAS-LN score (p < 0.001*) was identified as a significantly independent predictive factor for residual LN after operation.

**Conclusion:**

Clinically significant improvement in LN was observed in the majority of patients within 6 months after LDS, and the improvement of VAS-LN was slower than the VAS-LP. High pre-operative VAS-LN score can independently predict the presence of residual LN after surgery at 12-month follow up.

## Background

Pain, numbness, weakness and sensory disturbance of legs are the common symptoms of lumbar degenerative diseases (LDD) [1–3]. Lumbar decompression surgery (LDS) is the first choice for LDD once the conservative treatment failed [4,5]. The majority of patients who underwent LDS recovered well in leg pain (LP), and had a relatively high degree of satisfaction [6]. However, many patients still concerned about the residual numbness or paresthesia after the operation [7,8].

Postoperative residual leg numbness (LN) may be an important influencing factor leading to postoperative dissatisfaction [6]. Most studies focused on the improvement of LP instead of numbness after LDS, while the recovery of numbness is seldom studied [9]. Besides, there were few prospective studies to evaluate the factors that affect the recovery of sensory disturbance and LN after lumbar surgery [3,10−13]. The purpose of this study is to evaluate the changes of LN after LDS and to find the predictive factors for the recovery of LN.

## Methods

This study received approval from the Ethical Committee of the Clinical Medical College of Yangzhou University and all included patients were informed consent. A prospective cohort study was conducted, and patients undergoing LDS were enrolled at our institution between August 2020 and July 2021. The inclusion criteria were as follows: Patients who were scheduled to undergo LDS, with or without fusion for LDD due to radicular pain, numbness or neurogenic claudication symptoms; symptoms that were consistent with preoperative images; with no significant relief of symptoms after 6 weeks of regular conservative treatment. The exclusion criteria included pathological changes of lumbar vertebra, such as trauma, tumor, infection and those who needed one-stage decompression of other related cervical and/or thoracic lesions at the same time, a history of polyneuropathy or arteriosclerosis obliterans. After obtaining written informed consent, 317 consecutive patients with an average age of 57.4 years participated in this study. All patients underwent X rays, magnetic resonance imaging (MRI) and computed tomography (CT) examination before operation. To evaluate possible predictive factors affecting the recovery of leg symptoms, the following data were collected from all participants before surgery: medical history (hypertension, diabetes), duration of LP, duration of LN, body mass index (BMI), operation level, visual analogue score for leg pain (VAS-LP), visual analogue score for leg numbness (VAS-LN), oswestry disability index (ODI). Residual LN was defined as a patient with VAS-LN score≥1 at 1 year after surgery. Surgical indications for PLIF: Formal conservative treatment failed, patients with lumbar disc herniation which was multilevel, or giant disc herniation with bilateral lower limb symptoms or with lumbar instability, deformity; stenosis which has objective signs of nerve damage, typical symptoms of intermittent claudication, or with lumbar instability or spondylolisthesis; unilateral lumbar disc herniation or lumbar spinal stenosis with lumbar spondylolysis; lumbar disc herniation with large calcification; lumbar spondylolisthesis. Surgical indications for posterior transforaminal endoscopic discectomy: unilateral lumbar disc herniation or lateral recess stenosis of the L5-S1 segment. Surgical indications for oblique transforaminal endoscopic discectomy: unilateral lumbar disc herniation or lateral recess stenosis of the L4-5 segment.

## Outcome measurement

In this study, since there is no available scale for measuring numbness, we use VAS score to evaluate the degree of LN [14–16]. The functional disability was evaluated using the ODI score, where 0 indicates no disability and 50 indicates extreme disability. The VAS scores were from 0 (asymptomatic) to 10 (the worst symptom). Clinical data included duration of preoperative symptoms, length of hospital stay, VAS-LN, VAS-LP, ODI scores for evaluating preoperative symptoms. These outcomes were assessed preoperatively and at 3 months, 6 months and 12 months postoperatively.

### Statistical analysis

All the data were analyzed using the SPSS (version 19, IBM, USA). For qualitative variables, the total number and percentage of patients were provided. While for quantitative variables, the mean value and standard deviation (SD) were presented. Statistical analysis was performed with analysis of Wilcoxon signed rank sum test to evaluate changes in the VAS-LP, VAS-LN and ODI scores measured preoperatively and postoperatively. We used the univariate logistic regression analyses to calculate odds ratios (ORs) and 95% confidence intervals (CIs) in order to find out the predictive factors related to residual leg symptoms 12 months after operation. Significant items in univariate logistic regression analysis are further added to multivariate analysis. A P value of < 0.05 was used as statistical significance and the item was taken as the criterion for covariates being significant predictive factor. To reveal the relationship between different symptoms like LP, LN and disability, correlations between preoperative VAS and the ODI scores were analyzed by the Pearson correlation analysis.

## Result

### Cohort demographics characteristics and Surgical Procedures

During the follow-up period, one patient died in an accident and two patients were lost. A total of 314 patients were evaluated finally (follow-up rate: 99.05%). The 314 enrolled patients included 136 males and 178 females, aged from 21 to 83 years old, with an average age of 57.48 ± 13.27 years old. Patient’s demographic and clinical data are listed in (Table 1).

**Table 1:** Characteristics of the Study Population

No. of patients	314
**Age, mean** ± **SD (range)**	57.48 ± 13.27 (21–83)
**Sex, male/female**	136/178
**BMI, mean** ± **SD (range)**	24.47 ± 2.77 (19.10-33.13)
**Medical history, n (%)**	
Diabetes mellitus	33 (10.5%)
Hypertension	102(32.5%)
Previous spinal surgery	0 (0%)
**Symptoms**	
Cases of LP, n	314
Cases of LP and LN, n	250
Duration of LP, mean ± SD (range)	30.0 ± 49.2 (0.03–240)
Duration of LN, mean ± SD (range)	15.0 ± 34.8 (0-240)
Muscle weakness, n	3

Surgical decompression was performed for 365 segments of the 314 patients, with the most frequently operated levels located at L4–L5 (53.82%) and L5–S1 (26.75%), and only 14.65% of patients underwent multilevel procedures. Among all the cases, 203 patients (64.65%) underwent PLIF, and 111 patients (35.35%) underwent percutaneous foraminal endoscopic discectomy, among which 39 (35.14%) were posterior approach and 72 (64.86%) were lateral approach. The characteristics of surgical procedures were listed in (Table 2).

**Table 2:** surgical procedures

Level of surgery, n (%)		
	L2-3	3 (0.96%)
	L3-4	12 (3.82%)
	L4-5	169 (53.82%)
	L5-S1	84 (26.75%)
	Multiple levels	46 (14.65%)
Surgical approaches, n (%)		
	MIS procedure	111 (35.35%)
	PLIF	203 (64.65%)

### Clinical outcomes

**Fig. 1:**
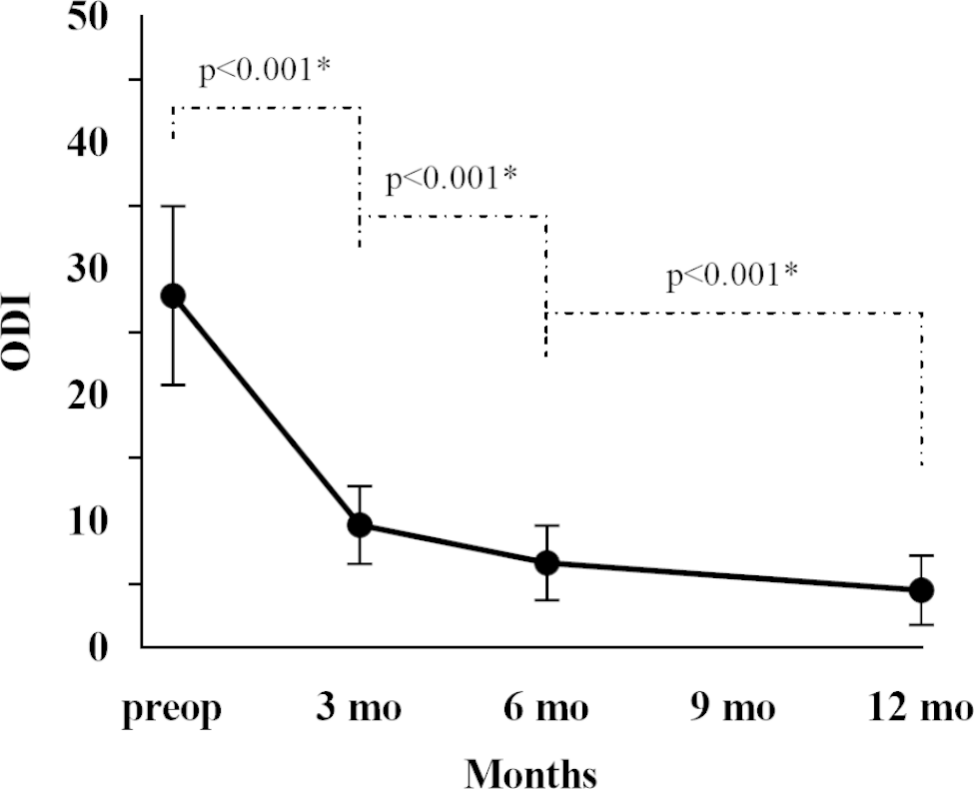
ODI change during the preoperative and postoperative visits at 3 months, 6 months, and at 12 months after surgery. *Indicated significant difference

LP was the most common symptom, followed by LN and weakness. Before operation, 250 patients (79. 6%) had both LN and LP, while only 64 patients were without LN. The clinical outcomes are shown in (Table 3). The preoperative mean VAS-LN score was 3.49 ± 2.44, which decreased to 1.91 ± 1.30 at 3 months (P < 0.001), to 1.29 ± 0.97 at 6 months (P < 0.001, compared with scores measured at 3 months postoperatively) and to 1.26 ± 0.96 at 12 months (P = 0.083, compared with scores measured at 6 months postoperatively) after surgery. The preoperative mean VAS-LP score was 6.05 ± 1.30, which decreased to 2.00 ± 0.86 at 3 months (P < 0.001), to 1.02 ± 0.80 at 6 months (P < 0.001, compared with scores measured at 3 months postoperatively), and to 0.49 ± 0.71 at 12 months (P = 0.083, compared with scores measured at 6 months postoperatively) after surgery. The preoperative mean ODI score was 27.90 ± 7.08, which decreased to 9.73 ± 3.09 at 3 months postoperatively (P < 0.001), to 6.72 ± 2.98 at 6 months postoperatively (P < 0.001, compared with scores measured at 3 months postoperatively) and to 4.57 ± 2.76 at 12 months postoperatively (P < 0.001 compared with scores measured at 6 months postoperatively, Fig. 1).

**Table 3:** Clinical outcomes

Variables	Preoperation (A)	3 months (B)	6 months (C)	12 months (D)	P value (A vs. D)
VAS-LP	6.05 ± 1.30	2.00 ± 0.86	1.02 ± 0.80	0.49 ± 0.71	P < 0.001
VAS-LN	3.49 ± 2.44	1.91 ± 1.30	1.29 ± 0.97	1.26 ± 0.96	P < 0.001
ODI	27.90 ± 7.08	9.73 ± 3.09	6.72 ± 2.98	4.57 ± 2.76	P < 0.001

**Fig. 2:**
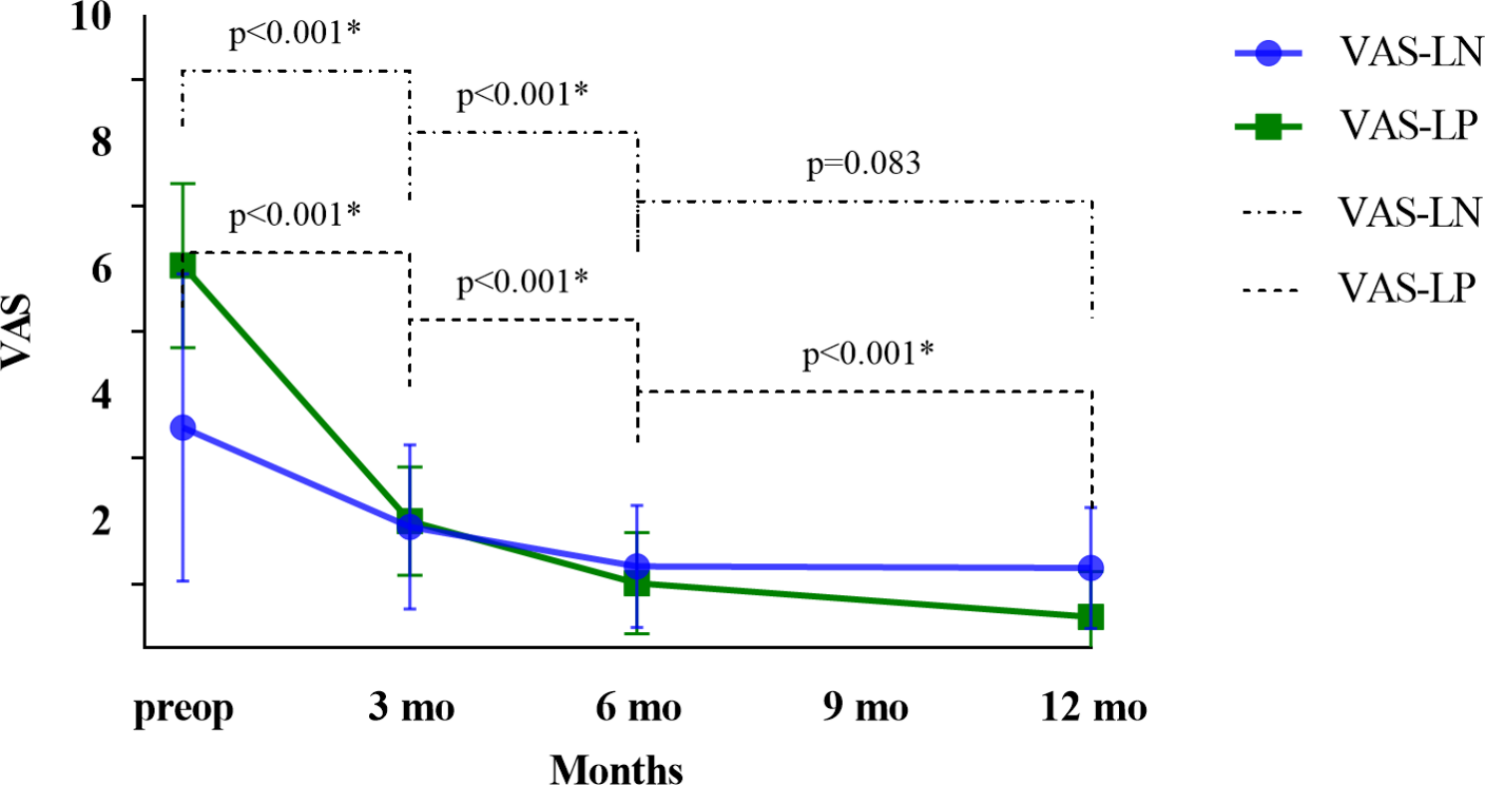
VAS-LN and VAS-LP change during the preoperative and postoperative visits at 3 months, 6 months, and at 12 months after surgery. *Indicated significant difference

The difference in the VAS-LN and VAS-LP scores between the preoperative and 12 month-postoperative values was 3.8 ± 3.4 and 4.3 ± 3.1, respectively. The change in the VAS-LN score was significantly smaller than that in the VAS-LP score **(**Fig. 2). We analyzed the correlation between post-op VAS-LN and ODI scores, the Pearson correlation analysis revealed a significant positive correlation between post-op VAS-LN and ODI (coefficient = 0.511, P < 0.001).

## Predictive factors for residual leg symptoms after surgery

At 12 months after surgery, 78 patients had no residual LN, and 236 patients (75.0%) still had residual LN (VAS-LN ≥ 1). Binary logistic regression was performed to evaluate the effects of demographic and clinical parameters on the improvement of LN at 12 months postoperatively (Table 4). Univariate analyses revealed that of the 12 predictor variables tested, 4 were statistically significant the predictive factors for residual LN, including age, duration of LN, preoperative VAS-LN and preoperative ODI score. While diabetes, hypertension, sex, BMI, no. of levels and surgical procedure were not the predictive factors for residual LN. Further, multivariate analyses revealed that significant independent predictive factors for residual LN were preoperative VAS-LN score (Table 4). The higher the preoperative VAS-LN score, the less likely it is to improve LN.

**Table 4:** Logistic regression analyses of predictive factors of residual leg numbness at 12 months after surgery

	Univariate analyses				Multivariate analyses		
Variables	OR	95%CI	P value		OR	95%CI	P value
Sex	1.302	0.526–3.227	0.568				
Age	1.047	1.011–1.085	0.010*		1.062	0.926–1.116	0.086
BMI	0.850	0.720–1.004	0.055				
Duration of leg numbness	1.202	1.023–1.412	0.026*		1.017	0.926–1.116	0.727
Duration of leg pain	0.998	0.990–1.007	0.672				
Preoperative VAS-LN	6.824	3.023–15.4.4	< 0.001*		0.942	2.735–18.432	< 0.001*
Preoperative VAS-LP	1.301	0.908–1.865	0.151				
Preoperative ODI	1.085	1.013–1.163	0.020*		0.942	0.780–1.137	0.532
Diabetes	0.272	0.033–2.235	0.226				
Hypertension	2.625	0.894–7.704	0.079				
No. of levels	0.717	0.186–2.771	0.630				
Open procedure	0.682	0.275–1.693	0.409				

## Discussion

Although many patients still suffer from residual LN after LDS, it has been rarely studied yet. The present study revealed that the greatest improvement in the post-operation VAS-LN, VAS-LP, ODI scores were observed during the initial 3 months. In addition, the VAS-LP, VAS-LN and ODI scores were also significantly improved at 6 months compared with scores measured at 3 months postoperatively. There were no significant changes in the VAS-LN score thereafter, while the VAS-LP and ODI scores were significantly improved at 12 months compared with scores measured at 6 months postoperatively.

“Numbness” is often used to describe various symptoms, including the feeling of walking on cobblestones or the feeling that the soles of the feet are surrounded by socks, paresthesia, numbness or loss of sensation [11]. Patients who underwent LDS often complained that pain intensity was obviously relieved, but LN still remained [6]. In the absence of a unified definition of residual leg numbness after surgery, we defined it as VAS-LN ≥ 1 at 1 year after surgery. Some patients even felt that the numbness of limbs was worse than before, which may lead to the compromise of patient satisfaction [6,10]. In this study, we revealed that LN in majority patients was significantly relieved within 6 months after surgery, and preoperative LN intensity was the only predictor for the presence of residual LN at 12 months follow up.

Our study showed that the improvement of VAS-LN score was worse than that of VAS-LP score after surgery. This finding was consistent with the outcomes reported by Huang et al. [17,18]. Besides, we revealed that VAS-LN scores continued to decrease within the first 6 months postoperatively, whereas there was no significant change thereafter. This outcome is different from another study which showed that the improvement of numbness was the most significant two weeks after operation, but there was no significant difference thereafter [18]. We underwent correlation analysis revealed a significant positive correlation between post-op VAS-LN and ODI. However, although there was a direct positive correlation, the correlation is small, and the clinical reference significance was limited.

Sensory nerves are composed of the Aβ, Aδ and C fibers. Aβ fibers refer to thick myelinated fibers, having the highest conduction velocities, while Aδ fibers refer to thin myelinated fibers, having intermediate conduction velocities, and C fibers refer to unmyelinated fibers, having the lowest conduction velocities [19]. Large nerve fibers with myelin sheath (such as Aδ fibers) can transmit touch pressure sensation and fast pain [19]. C fiber transmits pain and temperature sense. Previous clinical studies reported that numbness and paresthesia are related to the injury of large myelinated nerve fibers, the function of unmyelinated nerve fibers improved significantly within 6 weeks after operation, but the function of myelinated nerve fibers did not improve significantly within 12 months after operation [20, 21]. The recovery of pain and sensory disturbance transmitted by small unmyelinated nerve fibers (such as C fibers) were reported to be faster and more complete than myelinated nerve fibers (such as A-delta) involved in LN and pain symptoms [20, 22]. These conclusions are consistent with our findings that the improvement in the VAS-LN score was worse than that in the VAS-LP score and the improvement of LN was much slower than LP after LDS.

Pathophysiologically, the symptoms of nerve root injury caused by early compression may be related to mechanisms such as chemical inflammation and mechanical compression. Stimulation of inflammatory factors is likely to cause nerve root edema [3]]. Olmarker et al. [23] found that the intrinsic vasculature of the nerve roots in the cauda equina was very sensitive to mechanical compression. With the extension of compression time, the lumbar nerve root may undergo structural changes, resulting in focal demyelination. Yoshizawa et al. [24] studied the effect of chronic compression of lumbar nerve roots in a dog model. After three months of compression a fallout in the large myelinated fiber population and increase of thinly myelinated fibers were seen in and around the peripheral part of the nerve root. This suggests that the symptom of numbness and compression lasts longer, which may cause more irreversible nerve damage and slower recovery from numbness.

In previous studies, the predictors of residual numbness after LDS have not been sufficiently studied [3,17,18,25]. Huang et al. [18] reported that patients with LN lasting < 6 months preoperative experienced faster recovery. [18] In another study, Hiroki et al. [17]demonstrated that Patients with a longer duration of preoperative symptoms and a narrow preoperative dural sac cross-sectional area demonstrated worse improvement of LN. Via binary logistic regression, we found that only high preoperative LN scores may predict the presence of residual LN.

Therefore, to sum up, under the condition of giving informed consent before surgery, surgeons can explain the influencing factors of postoperative numbness recovery to patients, better guide patients to understand the expected results of postoperative recovery, and avoid patients’ insufficient understanding of postoperative numbness recovery, thereby resulting in better postoperative satisfaction.

### Limitations

There are several limitations in this study. First, we used the VAS score to evaluate residual LN after LDS. It is difficult to define the symptoms of numbness, patients may describe paresthesia and insensitivity as numbness. It is difficult to distinguish these symptoms by VAS scale. Second, we only followed up for one year, the follow-up time is relatively short, so we need a longer follow-up in the later period to understand the long-term prognosis of residual numbness.

## Conclusion

In conclusion, LN improves after LDS in the majority of patients, and the improvement of the VAS-LN score was slower than that of the VAS-LP score. LN improved significantly within the 6 months after surgery, and no significant change was noticed thereafter. Higher pre-operative VAS-LN scores were associated with the presence of residual LN. This information could be used to better consent and better manage expectations in patients undergoing LDS.

## Abbreviations

LN: leg numbness
LP: leg pain
LDS: lumbar decompression surgery
LDD: lumbar degenerative diseases
VAS: visual analog scale
ODI: oswestry disability index
VAS-LP: visual analogue score for leg pain
VAS-LN: visual analogue score for leg numbness
